# Critical Appraisal of Four IL-6 Immunoassays

**DOI:** 10.1371/journal.pone.0030659

**Published:** 2012-02-09

**Authors:** Dana K. Thompson, Kim M. Huffman, William E. Kraus, Virginia Byers Kraus

**Affiliations:** 1 Division of Rheumatology, Department of Medicine, Duke University Medical Center, Durham, North Carolina, United States of America; 2 Physical Medicine and Rehabilitation, Veterans Affairs Medical Center, Durham, North Carolina, United States of America; 3 Division of Cardiovascular Medicine, Department of Medicine, Duke University Medical Center, Durham, North Carolina, United States of America; University of California, San Francisco, United States of America

## Abstract

**Background:**

Interleukin-6 (IL-6) contributes to numerous inflammatory, metabolic, and physiologic pathways of disease. We evaluated four IL-6 immunoassays in order to identify a reliable assay for studies of metabolic and physical function. Serial plasma samples from intravenous glucose tolerance tests (IVGTTs), with expected rises in IL-6 concentrations, were used to test the face validity of the various assays.

**Methods and Findings:**

IVGTTs, administered to 14 subjects, were performed with a single infusion of glucose (0.3 g/kg body mass) at time zero, a single infusion of insulin (0.025 U/kg body mass) at 20 minutes, and frequent blood collection from time zero to 180 minutes for subsequent Il-6 measurement. The performance metrics of four IL-6 detection methods were compared: Meso Scale Discovery immunoassay (MSD), an Invitrogen Luminex bead-based multiplex panel (LX), an Invitrogen Ultrasensitive Luminex bead-based singleplex assay (ULX), and R&D High Sensitivity ELISA (R&D). IL-6 concentrations measured with MSD, R&D and ULX correlated with each other (Pearson Correlation Coefficients r = 0.47–0.94, p<0.0001) but only ULX correlated (r = 0.31, p = 0.0027) with Invitrogen Luminex. MSD, R&D, and ULX, but not LX, detected increases in IL-6 in response to glucose. All plasma samples were measurable by MSD, while 35%, 1%, and 4.3% of samples were out of range when measured by LX, ULX, and R&D, respectively. Based on representative data from the MSD assay, baseline plasma IL-6 (0.90±0.48 pg/mL) increased significantly as expected by 90 minutes (1.29±0.59 pg/mL, p = 0.049), and continued rising through 3 hours (4.25±3.67 pg/mL, p = 0.0048).

**Conclusion:**

This study established the face validity of IL-6 measurement by MSD, R&D, and ULX but not LX, and the superiority of MSD with respect to dynamic range. Plasma IL-6 concentrations increase in response to glucose and insulin, consistent with both an early glucose-dependent response (detectable at 1–2 hours) and a late insulin-dependent response (detectable after 2 hours).

## Introduction

Interleukin-6 (IL-6) is a cytokine that is released from a multitude of sites under a wide range of conditions. IL-6 secreted by immune cells, adipocytes and endothelial cells plays a well known role in the chronic low-grade inflammation characteristic of obesity [1], [2], diabetes and cardiovascular disease [3], as well as the acute immunological crises of infection and sepsis [4]. However, more recent studies have challenged the notion that the actions of IL-6 are either entirely immunological or wholly detrimental. IL-6 is released from contracting skeletal muscle before and after exercise, including moderate “non-damaging” exercise recommended by health professionals [5]. Furthermore, increases in plasma IL-6 concentrations directly stimulate both glucose [6] and lipid metabolism [7]. The additional finding that plasma IL-6 also rises in response to both acute hyperglycemic clamp and pulse [8], as well as hyperinsulinemia [9] highlights the potential role of this cytokine in substrate metabolism.

In normal healthy subjects free of inflammation, IL-6 concentrations are typically quite low, in the range of 0.2–7.8 pg/mL [10], [11] but can exceed concentrations of 1600 pg/mL in sepsis [12]. More modest increases in IL-6 concentrations are associated with age [13], hyperglycemia [8] and the physiologic stress of acute exercise [5]. As IL-6 is detectible in plasma it therefore has the potential to reflect systemic inflammatory, metabolic, and physiologic stimuli. To elucidate the multiple biological pathways in which IL-6 is involved, it is essential to have the ability to precisely quantify it across a broad dynamic concentration range and to have confidence in the face validity of the measure, *i.e.*, that it is measuring what it is purported to measure.

Therefore we designed a study to assess the performance metrics and face validity of cytokine concentrations generated by three different IL-6 immunoassays. In designing this study, we proposed several criteria *a priori* to judge the performance of each particular immunoassay. At a minimum, the dynamic range of the assay needed to be broad enough to measure both the low levels of IL-6 found in normal healthy individuals as well as the high levels characteristic of altered homeostasis associated with many disease or pre-disease conditions, ideally without the need for diluting samples to bring their values into range. Second, it was particularly important that assay reproducibility be high, not just with minimal variability within and between plates, but across different kit lots produced at different times in order to perform meta-analyses of data derived from multiple studies over time. Third, it was important that the values produced by a particular assay fulfill face validity criteria by showing the ability to detect potentially biologically relevant changes in plasma IL-6 in response to appropriate stimuli. This assessment required a sample set in which IL-6 concentrations would be expected to change in a predictable way in response to physiologic stimulation. Hyperglycemia [8] and hyperinsulinemia [9] have each been shown to raise plasma IL-6 levels, although with different response times. Therefore we chose to evaluate IL-6 in a set of serial samples obtained from healthy, but obese, middle-aged subjects during a frequently sampled intravenous glucose tolerance test (IVGTT). We hypothesized that single infusions of glucose and insulin would result in a measureable elevation in plasma IL-6 concentrations. Additionally, our goal was to identify a method for IL-6 quantification that met all three pre-specified assessment criteria.

## Methods

### Participants and Ethics Statement

On the basis of availability of sufficient volumes, samples from a total of 14 subjects were selected from the control arm (no exercise intervention) of the Studies Targeting Risk Reduction Interventions through Defined Exercise (STRRIDE) [14]. The purpose of STRRIDE was to assess the effect of the volume and intensity of exercise training on insulin sensitivity in a population of overweight, sedentary, non-diabetic, middle-aged adults. Informed written consent was obtained from all subjects, and all procedures were approved by the Institutional Review Board of Duke University Medical Center.

### Intravenous Glucose Tolerance Testing

The subjects underwent an IVGTT during which EDTA plasma was collected and stored at −80°C [14]. Briefly, glucose (50%) was injected into a catheter placed in the antecubital vein at a dose of 0.3 g/kg body weight, and insulin (0.025 U/kg body weight) was injected at minute 20. Blood samples were obtained frequently (T = 0, 2, 3, 4, 5, 6, 8, 10, 12, 14, 16, 19, 22, 25, 30, 40, 50, 60, 70, 80, 90, 100, 120, 140, 160, and 180 minutes), centrifuged, and plasma was frozen at −80°C for later analysis. Glucose and insulin were measured at all time points while IL-6 was measured at T = 0, 2, 6, 14, 19, 25, 30, 40, 60, 90, 120 and 180 minutes. A total of 162 samples were assayed using MSD, while limited plasma reduced the sample number to163 for R&D, 161 for Luminex, and 131 for HS Luminex.

### Analyte Measurement and Assay Validation

Plasma insulin was determined by immunoassay and glucose was determined with an oxidation reaction as previously described [15]. Plasma was assayed for IL-6 by four methods according to the manufacturers' protocols: 1) MSD - IL-6 Ultra Sensitive Assay (Meso Scale Discovery, Gaithersburg, Maryland), 2) R&D - High Sensitivity IL-6 ELISA (R&D Systems, Minneapolis, Minnesota), 3) ULX – IL-6 Ultrasensitive Singleplex Bead Kit (Invitrogen Corporation, Carlsbad, California), and 4) LX - as part of a Luminex Custom Multi-plex panel consisting of IL-6, and 12 other cytokines and chemokines: brain-derived neurotrophic factor (BDNF), granulocyte colony stimulating factor (GCSF), interleukin 1 receptor antagonist (IL-1RA) interleukins 1b, 2, and 8 (IL-1b, IL-2, IL-8), monocyte chemotactic protein 1 (MCP-1), regulated upon activation, normal T-cell expressed and secreted (RANTES), tumor necrosis factor a (TNFá), tumor necrosis factor receptors 1 and 2 (TNFR1 and TNFR2), and vascular endothelial growth factor (VEGF) (Invitrogen Corporation, Carlsbad California). In addition to IVGTT samples, each manufacturer's standards were assayed by each of the other immunoassay methods, with the exception of measurement of Luminex calibrators by Ultrasensitive Luminex, and vice versa, due to limiting reagents.

Pooled plasma from four healthy subjects served as a control specimen. For all assays, the mean of the pooled control sample plus or minus 2SDs was defined as the acceptable precision limits. Any plates in which the control falls outside of this range are repeated. No repeat plate analyses were required in this study based on this criterion. The dynamic range of each assay was defined by the highest and lowest concentrations of calibrators specified in each kit. Of note, the pooled control sample was within the manufacturer's published dynamic range for each assay (Table 1). The range of sample measurements was defined by the highest and lowest IL-6 concentrations in IVGTT plasma samples obtained via each method. Quantifiability, or the percentage of samples that were in the range of each assay, was defined as the ratio of the number of samples yielding concentrations within the assay range/total number of samples assayed; samples outside the measureable ranges were denoted as those that were either above or below the upper or lower limits of quantification. For purposes of graphical representation only, samples with IL-6 values above or below the range of detection were substituted with values twice the upper limit of quantification or one-half the lower limit of quantification, respectively, as determined by the highest and lowest concentrations of the standard curve.

**Table 1:** Performance metrics of four IL-6 immunoassays.

Assay Characteristic		MesoScale Discovery (MSD)	Invitrogen Multiplex Bead Panel (LX)	Invitrogen Ultrasensitive Singleplex (ULX)	R&D HS ELISA (R&D)
**Volume of Sample Required**		25 µl	50 µl	50 µl	100 µl
**Dynamic Range of Assay (pg/mL)**	Minimum	0.163	9.47	0.182	0.156
	Maximum	2500	6900	133	10
**Concentration of Control Samples (pg/mL)**	Mean (SD)	0.26 (0.05)	21.9 (5.3)	0.27 (0.03)	0.63 (0.01)
**Range of Sample Measurements (pg/mL0**	Minimum	0.3	9.47	0.43	0.31
	Maximum	13.7	384.9	23.99	8.89
	Mean	1.28	56.98	2.52	1.52
	Median	0.79	24.99	1.47	1.28
**Quantifiability of Samples**	% Samples in Assay Range	100% (163/163)	65% (104/161)	99% (130/131)	95.6% (155/162)
	% Samples <LLOQ	0	35% (57/161)	1% (1/131)	0.6% (1.162)
	% Samples >ULOQ	0	0	0	3.7% (6/162)
**Reproducibility (%CV)**	Intra-Plate Variability	4.8	5.6	18.3	6.3
	Inter-Plate Variability	15.7	24.3	28.1	17.9
	Inter-Lot Variability	19.9	37.2	NA	16.4
**Detection of Biological Response (Increase in IL-6 with Hyperglycemia**	Mean Difference (Stimulated – Baseline) (pg/mL)	3.35	3.42	5.02	8.32
	95% Confidence Interval	1.21, 5.48	5.9, 12.8	1.5, 8.5	3.54, 13.11
	Significance of Difference (Stimulated – Baseline)	P = 0.0048	P = 0.4445	P = 0.0088	P = 0.0024

%CV = 100*SD/Mean; LLOQ = lower limit of quantification; ULOQ = upper limit of quantification; NA not analyzed. Volume was limiting in some samples permitting the following numbers of independent analyses: MSD n = 163, R&D n = 162, LX n = 161, and ULX n = 131. Detection of a biological response was determined by paired t test of IL-6 concentrations measured at baseline and at 180 minutes.

Reproducibility was reported as percent coefficient of variation (%CV), calculated as 100*SD/Mean. Intra-plate variability was calculated using duplicate measure of manufacturers' calibrators and plasma IVGTT samples, based on availability as follows: for MSD, all calibrator curves and 163 IVGTT samples; for R&D, all calibrator curves and 162 IVGTT samples; for LX all calibrator curves (except ULX) and 22 IVGTT samples; and for ULX all calibrator curves except LX. Inter-plate and inter-lot variability was assessed using a pooled plasma sample (collected from four individuals) measured in duplicate on MSD and R&D and measurement of 100 beads from individual wells on LX and ULX. Two lots were compared for MSD and LX, three lots were compared for R&D, while only lot was available for ULX. To assess responsiveness of IL-6 to IVGTT, serum samples were measured in duplicate for MSD and R&D (unless sample was limited, as indicated) and in the case of the bead based assays, LX and ULX, a minimum of 100 beads were analyzed from individual wells.

Pearson correlation coefficients were calculated using IL-6 measurements from plasma samples. To assess agreement between assays we performed Bland Altman tests [16] of z score normalized data where z = (x – μ)/σ, where x is the raw concentration, and μ and σ are the mean and standard deviation of all concentrations for that assay. This was necessary due to the fact that the units of measure for LX were much greater than for the other assays. To evaluate responsiveness to glucose, untransformed IL-6 concentrations at t = 180 minutes were compared to baseline for each subject using the paired t test. In four subjects (1, 7, 8, and 13) baseline sample was unavailable for assay by ULX, therefore comparisons were made to sample collected at 2 or 6 minutes. Statistical analysis was performed using GraphPad Prism, with significance defined as p<0.05.

## Results

To assess the responsiveness of IL-6 to glucose and insulin, as well as to compare the performance parameters of three IL-6 detection methods, we used IVGTT plasma samples from 14 healthy subjects, of which 5 were women (36%), 3 were black (21%), the mean age was 50.4 yr (range 40–61), and mean BMI was 29.1 kg•m^−2^ (range 26.7–32.2). Numerous performance metrics were compared among the four immunoassays, as summarized in Table 1. Sample volumes required for assays varied from 25 µl (MSD) to 50 µl (LX and ULX) to 100 µl (R&D). For all IVGTT plasma samples, LX returned values that were significantly higher than those reported by the other three assays, while in most cases MSD returned the lowest values (Figures S1,S2,S3,S4,S5,S6,S7,S8,S9,S10,S11,S12,S13,S14). This rank order was also reflected in the overall means (pg/mL) of the IVGTT samples: LX (53.1), ULX (2.07), R&D (1.68), and MSD (1.08) (Table 1). IL-6 concentrations (mean, SD in pg/ml) of control samples pooled from the serum of four healthy subjects were reported highest by LX (21.9, 5.3) followed by R&D (0.63, 0.1), ULX (0.27, 0.03) and MSD (0.26, 0.05).

Reproducibility (both within plates and between plates and lots) was similar and acceptable for both MSD and R&D, but lower for LX and ULX. MSD, R&D, and ULX (but not LX) consistently detected changes in IL-6 concentration upon stimulation by glucose administered during the IVGTT (Table 1 and Figures S1,S2,S3,S4,S5,S6,S7,S8,S9,S10,S11,S12,S13,S14). Although similar in many respects, a notable difference between these assays was the dynamic range. While both R&D and ULX were designed to detect the very low levels of IL-6 typically found in healthy individuals, both assays failed to measure one low value (0.6% and 1% respectively). Additionally, R&D was constrained by an upper limit of 10 mg/ml, yielding out of range (too high) values for 6 (3.7%) samples. Although the upper range of the LX assay was presumably sufficient to capture these high values, it was constrained by a lower limit of detection of 9.47 pg/ml, thus failing to measure 35% of the samples assayed here. Only MSD detected IL-6 in all samples measured, and had the capacity to yield results on the first determination for samples with very high concentrations thus minimizing the need for sample dilution and reassay. Obtaining high-end measurements with R&D and ULX could potentially require dilution of samples, with the consequences of both higher costs (due to need for multiple repeat measurements) and higher technical variability in the studies.

**Figure 1:**
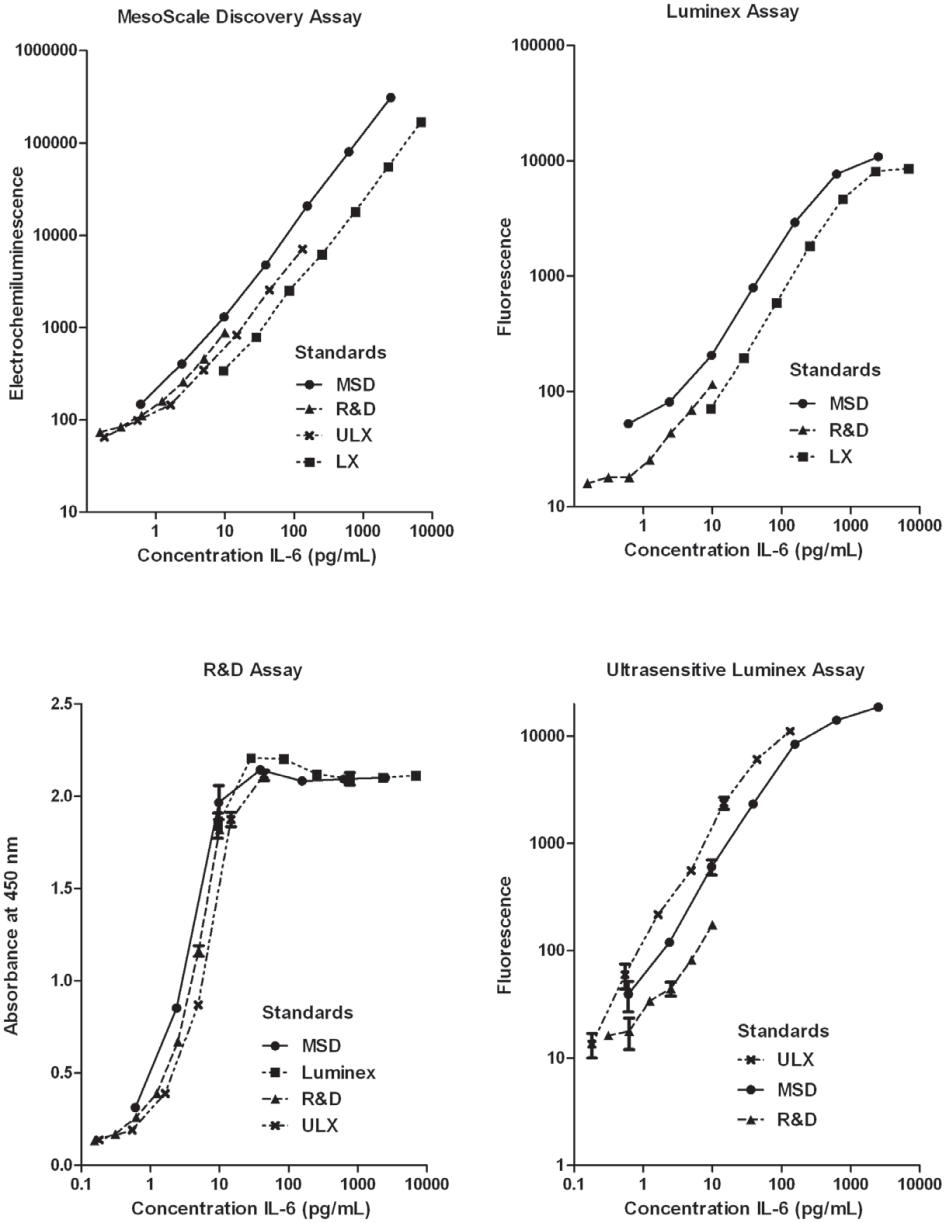
IL-6 protein calibrators of four immunoassays. IL-6 calibrators (standards) from MesoScale Discovery (• MSD), Invitrogen Luminex (▪ LX), and R&D High Sensitivity ELISA (▴ R&D), and Invitrogen Ultrasensitive Luminex (**×**ULX) were assayed using each of four manufacturers' kit components. Values represent duplicate measurement for MSD and R&D, and duplicate measures of at least 100 beads each for Luminex and Ultrasensitive Luminex.

Calibrators (IL-6 standards) from each manufacturer were tested on each of the other assays (Figure 1) and produced generally parallel standard curves. However, there was notable variability in the measured signals between different calibrators that were expected to contain similar concentrations of IL-6. This variability was generally least at low IL-6 concentrations and greater at higher concentrations. Variability between calibrators was lowest for R&D compared to the three other immunoassays, and highest for ULX. Additionally, the four assays displayed different rank orders of standard curves: MSD (MSD>R&D>ULX>LX); R&D (LX>MSD>R&D>ULX), LX (MSD>R&D>LX), and ULX (ULX>MSD>R&D).

**Figure 2:**
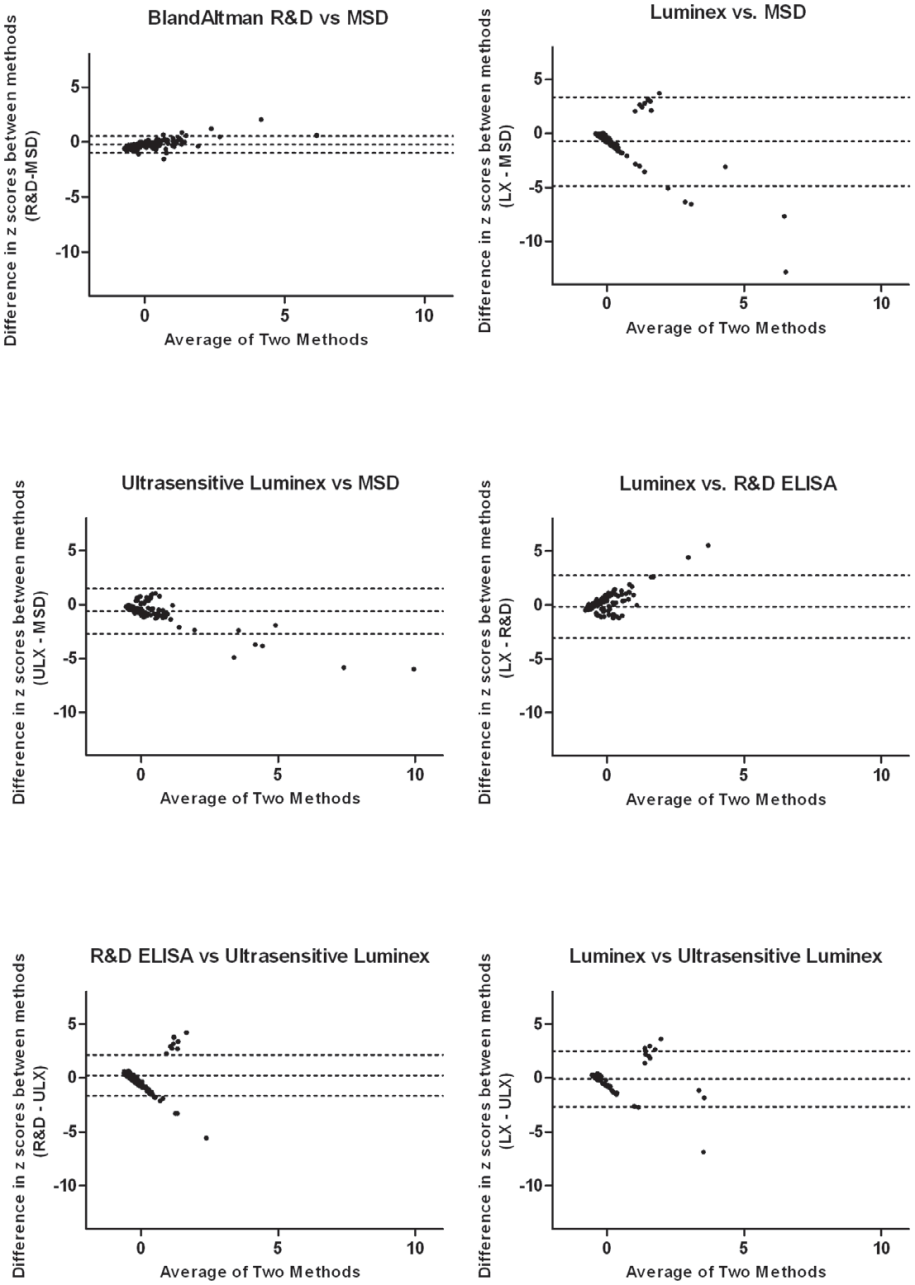
Modified Bland-Altman tests comparing four IL-6 immunoassays. **IL-6 concentrations**. IL-6 concentrations were standardized by calculating the z score using z = (x–μ)/σ, where x is the raw concentration, and μ and σ are the mean and standard deviation of all concentrations for that assay. The limits of agreement are denoted by hatch marks representing the mean ± 2SD of the differences in measurements. Out of range values (LX n = 57, ULX n = 1, R&D n = 7, as reported in Table 1) were excluded from these analyses.

Correlations of IL-6 concentrations (Table 2) between MSD and both R&D and ULX assays were strongest (Pearson correlation coefficient r = 0.94, p<0.0001; r = 0.90, p<0.0001, respectively), and weaker between R&D and ULX (r = 0.47, p<0.0001). LX correlated poorly with ULX (r = 0.31. p = 0.0027) and not at all with MSD and R&D (r = 0.15, p = 0.13; r = −0.17, p = 0.097). While Bland Altman plots (displaying the means vs. the differences of sample measurements) are the standard method of assessing agreement between assays, the hundreds-fold higher values returned by LX compared with the other three assays necessitated comparison across assays using z scores that normalize each set of values and express them as standard deviations from the mean. Bland Altman plots revealed the highest agreement (narrower limits of agreement) between MSD, R&D, and ULX, and essentially no agreement between LX and any of the other assays (Figure 2).

**Table 2:** Pearson Correlations between four IL-6 assays.

		MesoScale Discovery (MSD)	R&D HS ELISA (R&D)	Invitrogen Multiplex Bead Panel (LX)
R&D HS ELISA (R&D)	r	0.94		
	95% CI	0.92–0.96		
	p	<0.0001		
Invitrogen Multiplex Bead Panel (LX)	r	0.15	−0.17	
	95% CI	−0.04–0.339	−0.36–0.032	
	p	0.13	0.097	
Invitrogen Ultrasensitive Singleplex (ULX)	r	0.90	0.47	0.31
	95% CI	0.86–0.93	0.32–0.598	0.11–0.486
	p	<0.0001	<0.0001	0.0027

Pearson correlation coefficient, r; confidence interval, CI.

**Figure 3:**
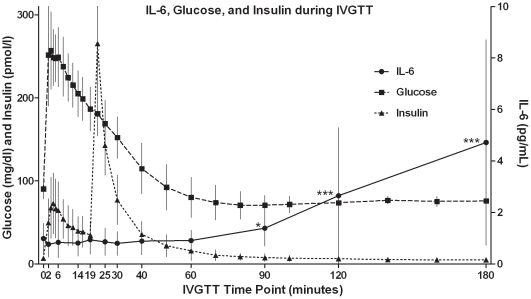
Plasma glucose, insulin, and IL-6 during frequently sampled intravenous glucose tolerance test (IVGTT). Plasma concentrations of glucose (▪) and insulin (▴) (defined by the left Y axis) and IL-6 as measured by Meso Scale Discovery (•) (defined by the right Y axis) are shown. Glucose (50%, 0.3 g/kg body mass) was infused at time zero, and insulin (0.025 U/kg body mass) was infused at 20 minutes. Measurements are mean (SD) for n = fourteen subjects; * p value<0.05; ** p value<0.01 as compared to baseline.

To assess the responsiveness of the IL-6 immunoassays to changes in IL-6, we measured IL-6 in serial plasma samples derived from IVGTTs. We hypothesized that an assay capable of detecting the modest changes in IL-6 concentrations would meet criteria for biological plausibility and face validity; namely that the assay would be capable of detecting biologically relevant variation in IL-6 concentrations under a wide range of conditions and would be measuring what it purports to measure. Over the course of the 180 minute IVGTT, IL-6 increased significantly compared to baseline, as detected by MSD, R&D, ULX, but not LX. These increases in IL-6 were discernible when the IVGTT time course of each individual subject was plotted (Figures S1,S2,S3,S4,S5,S6,S7,S8,S9,S10,S11,S12,S13,S14). To further characterize the concentrations of IL-6, the timecourse of mean glucose and insulin concentrations during the course of the IVGTT were plotted (Figure 3, representative data derived from MSD assay). As expected, mean glucose rose after glucose infusion peaking three minutes after the beginning of the IVGTT at a mean (SD) concentration of 253 (45.8) mg/dL before returning to baseline within one hour. Insulin, via endogenous release, increased immediately following glucose infusion, reaching an initial mean peak (SD) concentration of 72.3 (38.0) pmol/L at 4 minutes, and a subsequent peak mean (SD) concentration of 270.4 (99.5) pmol/L at 22 minutes following insulin infusion at 20 minutes, then returned to a concentration equivalent to baseline concentrations by 90 minutes. The mean (SD) IL-6 concentration, 0.90 (0.49) pg/mL, was equivalent to baseline concentration until 60 minutes after the start of the IVGTT, became significantly different from baseline at 90 minutes (1.29±0.59 pg/mL, p = 0.049) and continued to rise steadily until 180 minutes (4.25±3.67 pg/mL, p = 0.0048) when the IVGTT was terminated. These characteristic changes in IL-6 during an IVGTT confirm the face validity to the MSD immunoassay and the R&D and ULX assays that showed a similar pattern of IL-6 change.

## Discussion

In this validation study, we sought not only to quantify the dynamic range and reproducibility of each method, but importantly, to establish the face validity of the IL-6 immunoassays through demonstration of biologically plausible change during the course of an IVGTT. Based on results of Esposito et al. [8], and the fact that we used similar glucose pulse conditions, we expected to see increases in plasma IL-6 levels during the course of an IVGTT study. MSD, R&D and ULX all detected changes in IL-6 concentrations in response to glucose and insulin, and were comparable with regard to other assay metrics, with the exception that MSD had a broader dynamic range than ULX or R&D. Correlation of concentrations, and agreement as assessed by modified Bland-Altman tests were strong between the three assays but weak with LX, suggesting that the three assays are indeed measuring the same analyte, *i.e.*, IL-6. The variability in standard curves of different manufacturers (Figure 2), while not extreme, was nevertheless noteworthy. Some variability in concentrations and the potential presence of impurities in different formulations, even from the same manufacturer, are to be expected. Calibrator variation is likely to be the source of systematic bias in measurements between different assays, although differences in the recognizing antibodies may also play a role. These differences are to be expected since immunoassays are neither capable nor designed to yield absolutely precise concentrations of analytes and ultimately this variability could be adjusted with an international standard.

The IL-6 results derived from the IVGTTs suggested two phases of an IL-6 response that appear to reflect distinct but coordinated regulation by glucose and insulin. Furthermore, the extended duration of IL-6 elevation suggests that gene expression, protein synthesis and release, and clearance may all be involved in IL-6 regulation by glucose and insulin, demonstrating an interaction between immunological and metabolic pathways. In our study, both glucose and insulin were infused intravenously, the former at the start of the IVGTT and the latter after 20 minutes. Although the infusion of glucose and insulin in our study were episodic rather than continuous, and insulin was infused at lower concentrations than previously tested [9], we nevertheless detected a significant increase in plasma IL-6 concentrations in response to both stimuli. IL-6 rose steadily after the first 60 minutes through the end of the study at 3 hours, correlating temporally with both glucose and insulin infusion.

These data complement and expand the existing data on IL-6 variation in response to change in glucose homeostasis. In one previous study [8], hyperglycemic clamp (with inhibition of insulin release) led to a phasic (rapid rise by one hour, then return to baseline by three hours) response of IL-6. In contrast, sustained hyperinsulinemia (with glucose held at fasting levels) led to a slow and continuous rise in IL-6 beginning after 2–3 hours and continuing at least 6 hours [9]. It remains to be seen how changes in glucose tolerance and insulin sensitivity, as impaired by obesity or ameliorated by exercise, might be reflected in the pattern of IL-6 response.

As the number and type of molecular assays proliferate, it becomes increasingly important for research groups to thoughtfully choose and validate the methods by which they generate biomarker data. Earlier efforts may have dispensed with this step for the simple reason that only one assay may have been available for a particular analyte, but years of product development have increased options as well as the responsibility to deliberately select a method that optimizes the criteria required of the research objectives. Regarding the technical aspects of this study, of the three assay methods, the MSD Ultra Sensitive Immunoassay proved preferable for quantifying IL-6. The dynamic range accommodated both the very high and low concentrations, variability within and between plates and between lots was sufficiently low, and the assay required only small volumes of sample.. We experienced problems in measuring IL-6 with the Invitrogen Luminex assay in the context of a multiplex panel, including poor reproducibility (particularly between plates and between lots), and inconsistent detection of analyte changes in response to physiologic stimuli. We have had more consistent results and continue to use Invitrogen Luminex for other analytes. The Invitrogen Ultrasensitive Luminex provides an acceptable alternative, although its dynamic range and reproducibility are more limited than MSD. Perhaps most important, the face validity of the MSD, R&D, and ULX assays - *i.e.*, that they are actually measuring biologically relevant IL-6 concentrations - was established by both the correlation and agreement between the three assays) as well as the detection of changes in IL-6 concentrations in response to exogenous glucose and insulin, representing a biologically relevant stimulus. In conclusion, MSD, R&D and ULX all provided reliable assays with high face validity but only MSD, with its broad dynamic range, provided values in the linear range of the assay in the first determination, thereby minimizing cost, time expenditure and sample use.

## Supporting Information

Figure S1 **Plasma IL-6 during frequently sampled intravenous glucose tolerance test (IVGTT) in subject 1.** Plasma concentrations of IL-6 were measured by MesoScale Discovery (• MSD), R&D High Sensitivity ELISA (▪ R&D), Invitrogen Luminex (▴ LX) and Invitrogen Ultrasensitive Luminex (**×**ULX). Due to limited sample volumes, it was not possible to provide measurements for one time point (180 minutes) using LX, for one time point (120 minutes) using R&D, and for two time points (30 and 120 minutes) using ULX.
(TIF)

Figure S2 **Plasma IL-6 during frequently sampled intravenous glucose tolerance test (IVGTT) in subject 2.** Plasma concentrations of IL-6 were measured by MesoScale Discovery (• MSD), R&D High Sensitivity ELISA (▪ R&D), and Invitrogen Luminex (▴ LX) and Invitrogen Ultrasensitive Luminex (**×**ULX). No sample was available for measurement at one time point (14 minutes).
(TIF)

Figure S3 **Plasma IL-6 during frequently sampled intravenous glucose tolerance test (IVGTT) in subject 3.** Plasma concentrations of IL-6 were measured by MesoScale Discovery (• MSD), R&D High Sensitivity ELISA (▪ R&D), and Invitrogen Luminex (▴ LX) and Invitrogen Ultrasensitive Luminex (**×**ULX). One sample (180 minutes) returned an IL-6 value above the range of detection (R&D) and was substituted with a value twice the upper limit of quantification, as determined by the highest concentration of the standard curve, and denoted by (<). No sample was available for measurement at one time point (6 minutes).
(TIF)

Figure S4 **Plasma IL-6 during frequently sampled intravenous glucose tolerance test (IVGTT) in subject 4.** Plasma concentrations of IL-6 were measured by MesoScale Discovery (• MSD), R&D High Sensitivity ELISA (▪ R&D), and Invitrogen Luminex (▴ LX) and Invitrogen Ultrasensitive Luminex (**×**ULX). Due to limited sample volumes, it was not possible to provide measurements for two time points (6 and 25 minutes) using ULX.
(TIF)

Figure S5 **Plasma IL-6 during frequently sampled intravenous glucose tolerance test (IVGTT) in subject 5.** Plasma concentrations of IL-6 were measured by MesoScale Discovery (• MSD), R&D High Sensitivity ELISA (▪ R&D), and Invitrogen Luminex (▴ LX) and Invitrogen Ultrasensitive Luminex (**×**ULX). Due to limited sample volumes, it was not possible to provide measurements for one time point (6 minutes) using LX.
(TIF)

Figure S6 **Plasma IL-6 during frequently sampled intravenous glucose tolerance test (IVGTT) in subject 6.** Plasma concentrations of IL-6 were measured by MesoScale Discovery (• MSD), R&D High Sensitivity ELISA (▪ R&D), and Invitrogen Luminex (▴ LX) and Invitrogen Ultrasensitive Luminex (**×**ULX). Due to limited sample volumes, it was not possible to provide measurements for four time points (2, 14, 30, and 120 minutes) using ULX.
(TIF)

Figure S7 **Plasma IL-6 during frequently sampled intravenous glucose tolerance test (IVGTT) in subject 7.** Plasma concentrations of IL-6 were measured by MesoScale Discovery (• MSD), R&D High Sensitivity ELISA (▪ R&D), and Invitrogen Luminex (▴ LX) and Invitrogen Ultrasensitive Luminex (**×**ULX). Due to limited sample volumes, it was not possible to provide measurements for six time points (19, 30, 40, 60, 120, and 180 minutes) using ULX.
(TIF)

Figure S8 **Plasma IL-6 during frequently sampled intravenous glucose tolerance test (IVGTT) in subject 8.** Plasma concentrations of IL-6 were measured by MesoScale Discovery (• MSD), R&D High Sensitivity ELISA (▪ R&D), and Invitrogen Luminex (▴ LX) and Invitrogen Ultrasensitive Luminex (**×**ULX). Two samples returned IL-6 values above the range of detection (R&D) and were substituted with values twice the upper limit of quantification, as determined by the highest concentration of the standard curve and denoted by (<).
(TIF)

Figure S9 **Plasma IL-6 during frequently sampled intravenous glucose tolerance test (IVGTT) in subject 9.** Plasma concentrations of IL-6 were measured by MesoScale Discovery (• MSD), R&D High Sensitivity ELISA (▪ R&D), and Invitrogen Luminex (▴ LX) and Invitrogen Ultrasensitive Luminex (**×**ULX). Due to limited sample volumes, it was not possible to provide measurements for three time points (14, 19, and 90 minutes) using ULX.
(TIF)

Figure S10 **Plasma IL-6 during frequently sampled intravenous glucose tolerance test (IVGTT) in subject 10.** Plasma concentrations of IL-6 were measured by MesoScale Discovery (• MSD), R&D High Sensitivity ELISA (▪ R&D), and Invitrogen Luminex (▴ LX) and Invitrogen Ultrasensitive Luminex (**×**ULX). One sample (180 minutes) returned an IL-6 value above the range of detection (R&D) and was substituted with a value twice the upper limit of quantification, as determined by the highest concentration of the standard curve, and denoted by (<).
(TIF)

Figure S11 **Plasma IL-6 during frequently sampled intravenous glucose tolerance test (IVGTT) in subject 11.** Plasma concentrations of IL-6 were measured by MesoScale Discovery (• MSD), R&D High Sensitivity ELISA (▪ R&D), and Invitrogen Luminex (▴ LX) and Invitrogen Ultrasensitive Luminex (**×**ULX). One sample (180 minutes) returned an IL-6 value above the range of detection (R&D) and was substituted with a value twice the upper limit of quantification, as determined by the highest concentration of the standard curve, and denoted by (<). Due to limited sample volumes, it was not possible to provide measurements for one time point (14 minutes) using ULX.
(TIF)

Figure S12 **Plasma IL-6 during frequently sampled intravenous glucose tolerance test (IVGTT) in subject 12.** Plasma concentrations of IL-6 were measured by MesoScale Discovery (• MSD), R&D High Sensitivity ELISA (▪ R&D), and Invitrogen Luminex (▴ LX) and Invitrogen Ultrasensitive Luminex (**×**ULX).Due to limited sample volumes, it was not possible to provide measurements for four time points (6, 14, 40, and 60 minutes) using ULX.
(TIF)

Figure S13 **Plasma IL-6 during frequently sampled intravenous glucose tolerance test (IVGTT) in subject 13.** Plasma concentrations of IL-6 were measured by MesoScale Discovery (• MSD), R&D High Sensitivity ELISA (▪ R&D), and Invitrogen Luminex (▴ LX) and Invitrogen Ultrasensitive Luminex (**×**ULX). No sample was available for measurement at one time point (120 minutes).
(TIF)

Figure S14 **Plasma IL-6 during frequently sampled intravenous glucose tolerance test (IVGTT) in subject 14.** Plasma concentrations of IL-6 were measured by MesoScale Discovery (• MSD), R&D High Sensitivity ELISA (▪ R&D), and Invitrogen Luminex (▴ LX) and Invitrogen Ultrasensitive Luminex (**×**ULX). One sample (180 minutes) returned an IL-6 value above the range of detection (R&D) and was substituted with a value twice the upper limit of quantification, as determined by the highest concentration of the standard curve, and denoted by (<). No sample was available for measurement at two time points (19 and 60 minutes).
(TIF)

## References

[1] Fain JN (2010). Release of inflammatory mediators by human adipose tissue is enhanced in obesity and primarily by the nonfat cells: a review.. Mediators of Inflammation 2010.

[2] Galic S, Oakhill JS, Steinberg GR (2010). Adipose tissue as an endocrine organ.. Molecular and Cellular Endocrinology.

[3] Gustafson B (2010). Adipose tissue, inflammation and atherosclerosis.. Journal of Atherosclerosis and Thrombosis.

[4] Ventetuolo CE, Levy MM (2008). Biomarkers: diagnosis and risk assessment in sepsis.. Clinics in Chest Medicine.

[5] Pedersen BK, Febbraio MA (2008). Muscle as an endocrine organ: focus on muscle-derived interleukin-6.. Physiological Reviews.

[6] Glund S, Deshmukh A, Long YC, Moller T, Koistinen HA (2007). Interleukin-6 directly increases glucose metabolism in resting human skeletal muscle.. Diabetes.

[7] Wolsk E, Mygind H, Grondahl TS, Pederen BK, Hall Gv (2010). IL-6 selectively stimulates fat metabolism in human skeletal muscle.. American Journal of Physiology - Endocrinology and Metabolism.

[8] Esposito K, Nappo F, Marfella R, Giugliano G, Giugliano F (2002). Inflammatory Cytokine Concentrations Are Acutely Increased by Hyperglycemia in Humans: Role of Oxidative Stress.. Circulation.

[9] Krogh-Madsen R, Plomgaard P, Keller P, Keller C, Pedersen BK (2003). Insulin stimulates interleukin-6 and tumor necrosis factor-á gene expression in human subcutaneous adipose tissue.. American Journal of Physiology - Endocrinology and Metabolism.

[10] Bas S, Gauthier BR, Spenato U, Gabay SSC (2004). CD14 is an acute-phase protein.. Journal of Immunology.

[11] Yoshida N, Ikemoto S, Narita K, Sugimura K, Wada S (2002). Interleukin-6, tumor necrosis factor á and interleukin-1 â in patients with renal cell carcinoma.. British Journal of Cancer.

[12] Martin H, Olander B, Norman M (2001). Reactive hyperemia and interleukin 6, interleukin 8, and tumor necrosis factor-áin the diagnosis of early-onset neonatal sepsis.. Pediatrics.

[13] Wei J, Xu H, Davies JL, Hemmings GP (1992). Increase of plasma IL-6 concentration with age in healthy subjects.. Life Sciences.

[14] Huffman KM, Shah SH, Stevens RD, Bain JR, Muehlbauer M (2009). Relationships between circulating metabolic intermediates and insulin action in overweight to obese, inactive men and women. .. Diabetes Care.

[15] Houmard JA, Tanner CJ, Slentz CA, Duscha BD, McCartney JS (2004). Effect of the volume and intensity of exercise training on insulin sensitivity.. Journal of Applied Physiology.

[16] Altman DG, Bland JM (1983). Measurement in medicine: the analysis of method comparison studies.. The Statistician.

