# Supplementary figures and images for: Critical Appraisal of Four IL-6 Immunoassays
Source: PLoS One. 2012 Feb 9;7(2):e30659. doi: 10.1371/journal.pone.0030659 (PMC3276568; doi:10.1371/journal.pone.0030659)

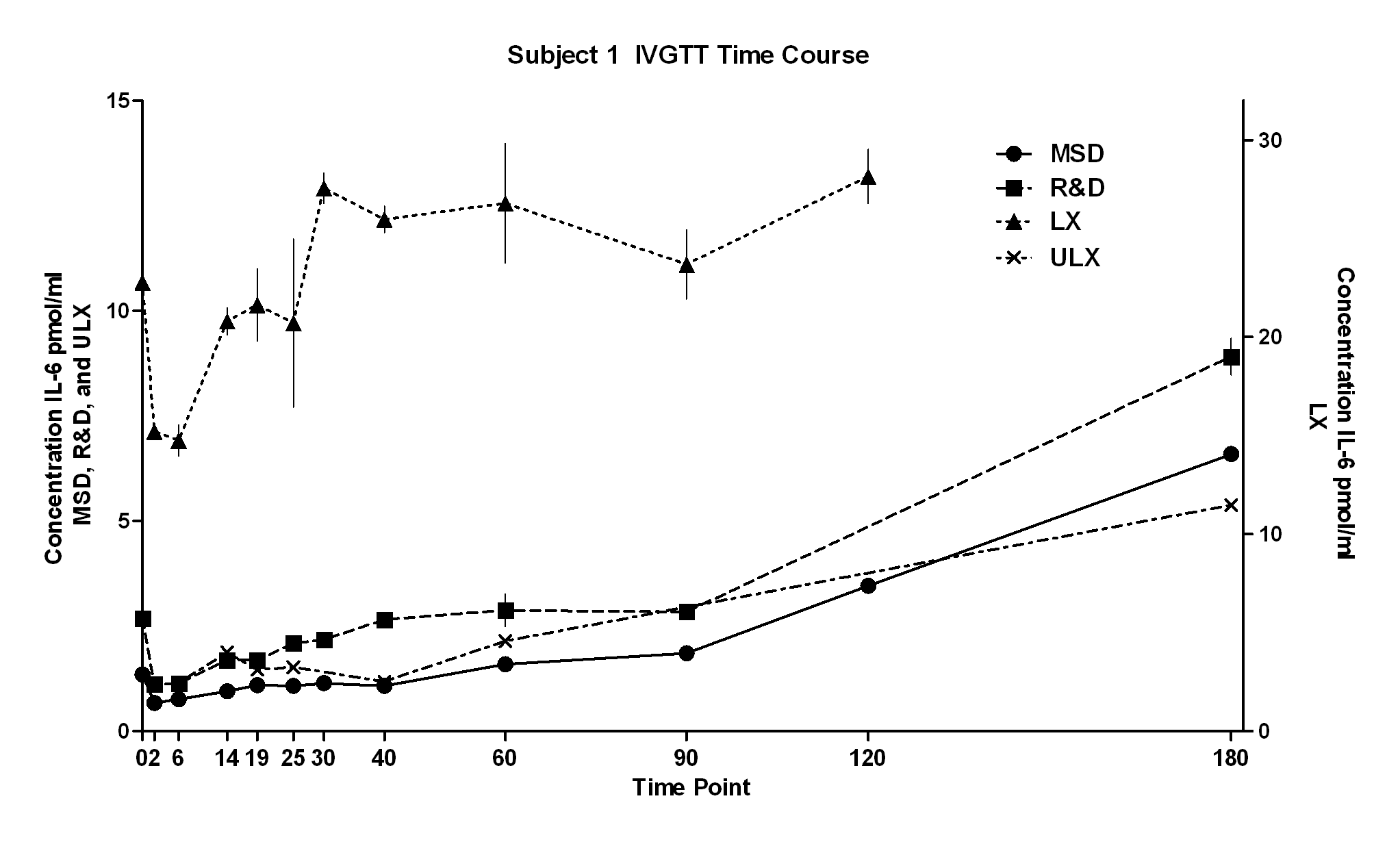

Supplement: Figure S1 — Plasma IL-6 during frequently sampled intravenous glucose tolerance test (IVGTT) in subject 1. Plasma concentrations of IL-6 were measured by MesoScale Discovery (• MSD), R&D High Sensitivity ELISA (▪ R&D), Invitrogen Luminex (▴ LX) and Invitrogen Ultrasensitive Luminex (×ULX). Due to limited sample volumes, it was not possible to provide measurements for one time point (180 minutes) using LX, for one time point (120 minutes) using R&D, and for two time points (30 and 120 minutes) using ULX. (TIF) [file pone.0030659.s001.tif]

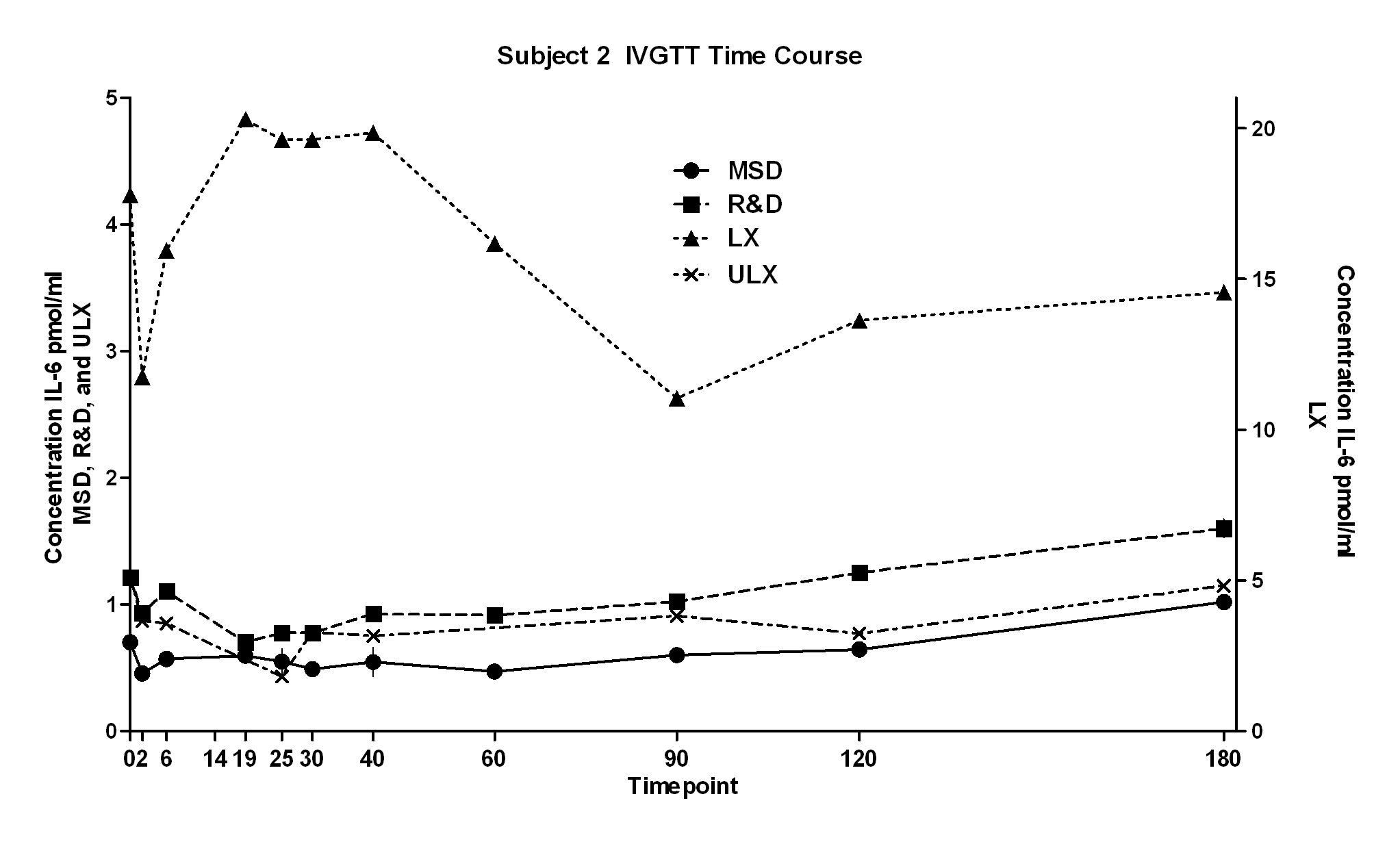

Supplement: Figure S2 — Plasma IL-6 during frequently sampled intravenous glucose tolerance test (IVGTT) in subject 2. Plasma concentrations of IL-6 were measured by MesoScale Discovery (• MSD), R&D High Sensitivity ELISA (▪ R&D), and Invitrogen Luminex (▴ LX) and Invitrogen Ultrasensitive Luminex (×ULX). No sample was available for measurement at one time point (14 minutes). (TIF) [file pone.0030659.s002.tif]

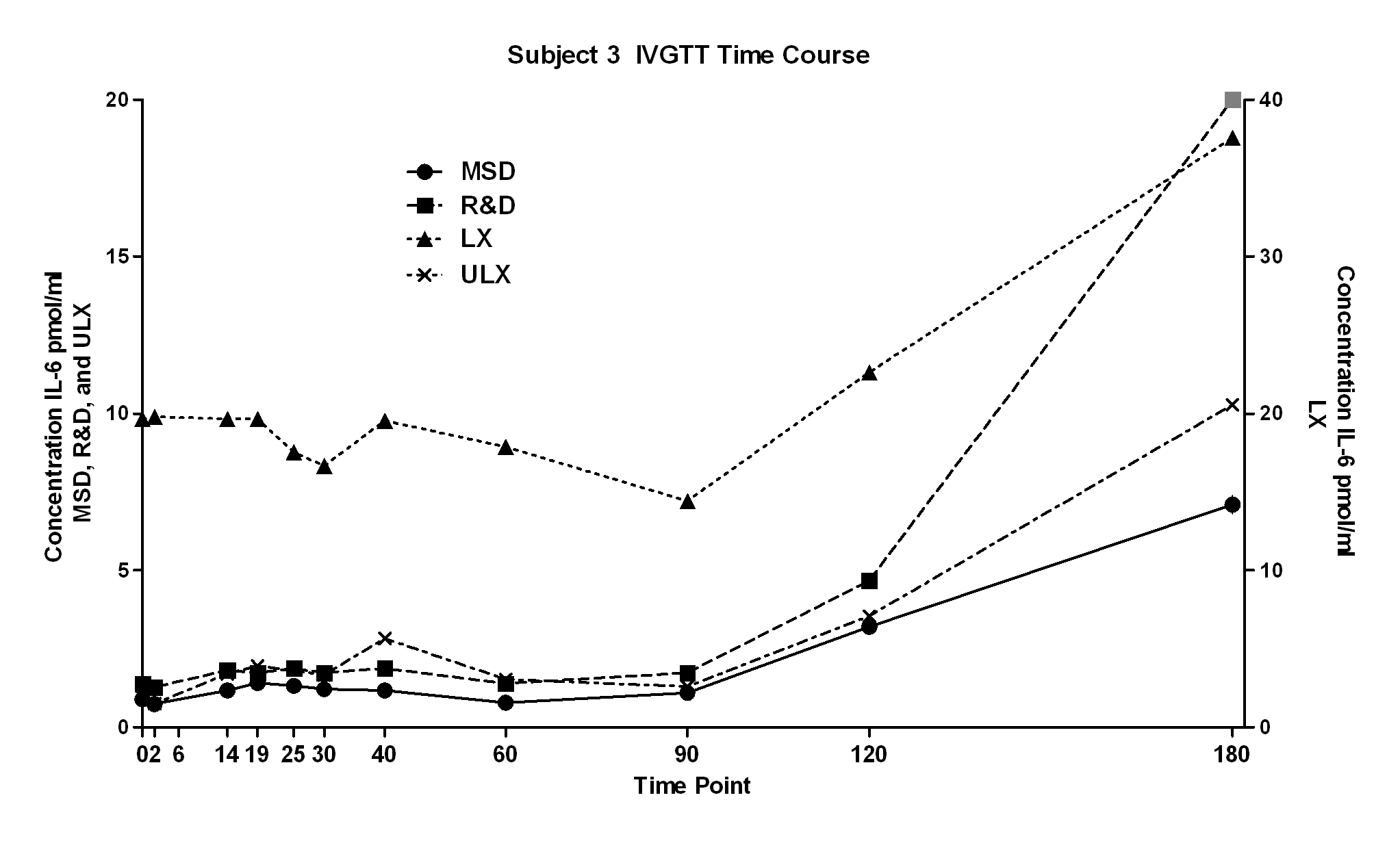

Supplement: Figure S3 — Plasma IL-6 during frequently sampled intravenous glucose tolerance test (IVGTT) in subject 3. Plasma concentrations of IL-6 were measured by MesoScale Discovery (• MSD), R&D High Sensitivity ELISA (▪ R&D), and Invitrogen Luminex (▴ LX) and Invitrogen Ultrasensitive Luminex (×ULX). One sample (180 minutes) returned an IL-6 value above the range of detection (R&D) and was substituted with a value twice the upper limit of quantification, as determined by the highest concentration of the standard curve, and denoted by (<). No sample was available for measurement at one time point (6 minutes). (TIF) [file pone.0030659.s003.tif]

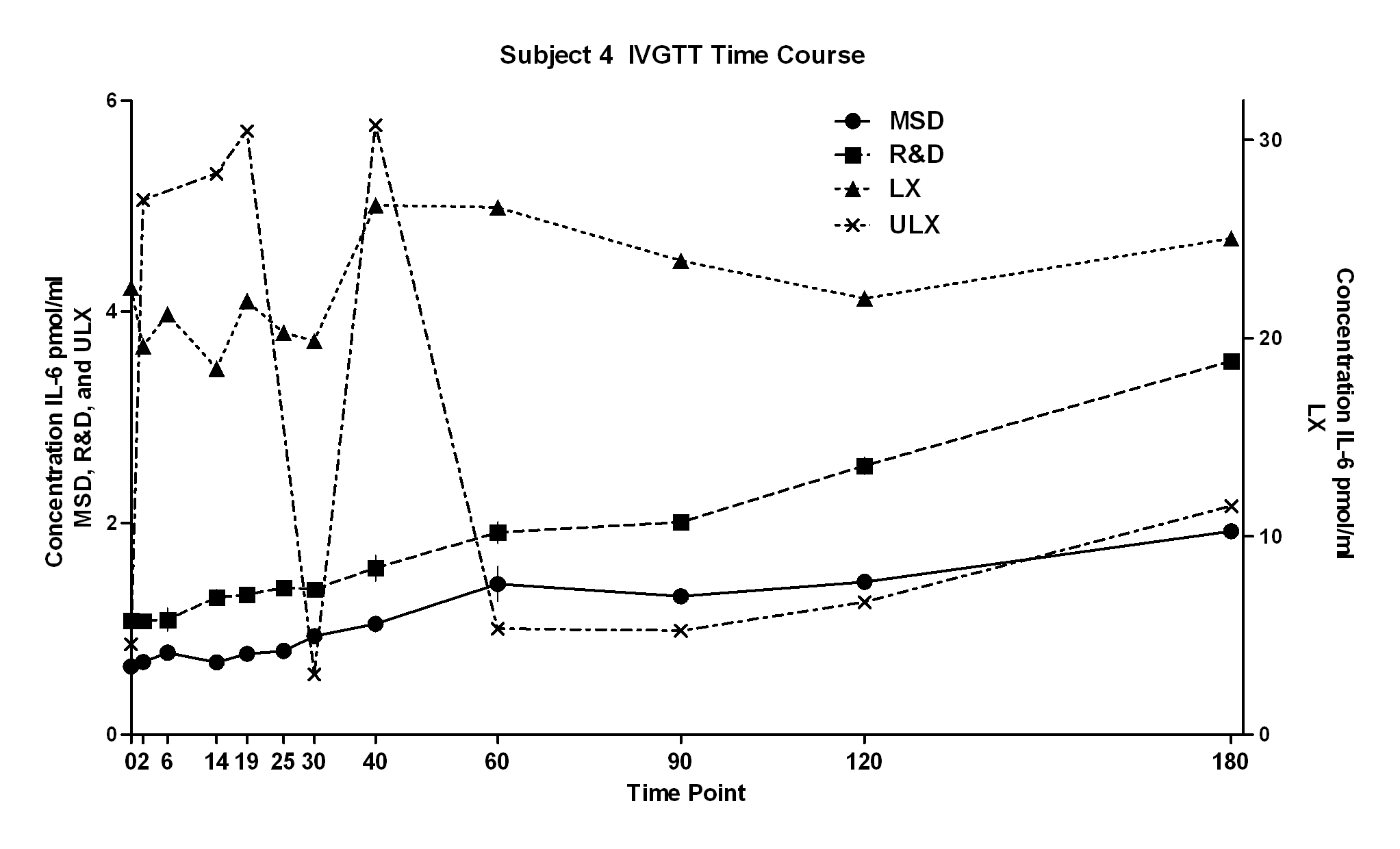

Supplement: Figure S4 — Plasma IL-6 during frequently sampled intravenous glucose tolerance test (IVGTT) in subject 4. Plasma concentrations of IL-6 were measured by MesoScale Discovery (• MSD), R&D High Sensitivity ELISA (▪ R&D), and Invitrogen Luminex (▴ LX) and Invitrogen Ultrasensitive Luminex (×ULX). Due to limited sample volumes, it was not possible to provide measurements for two time points (6 and 25 minutes) using ULX. (TIF) [file pone.0030659.s004.tif]

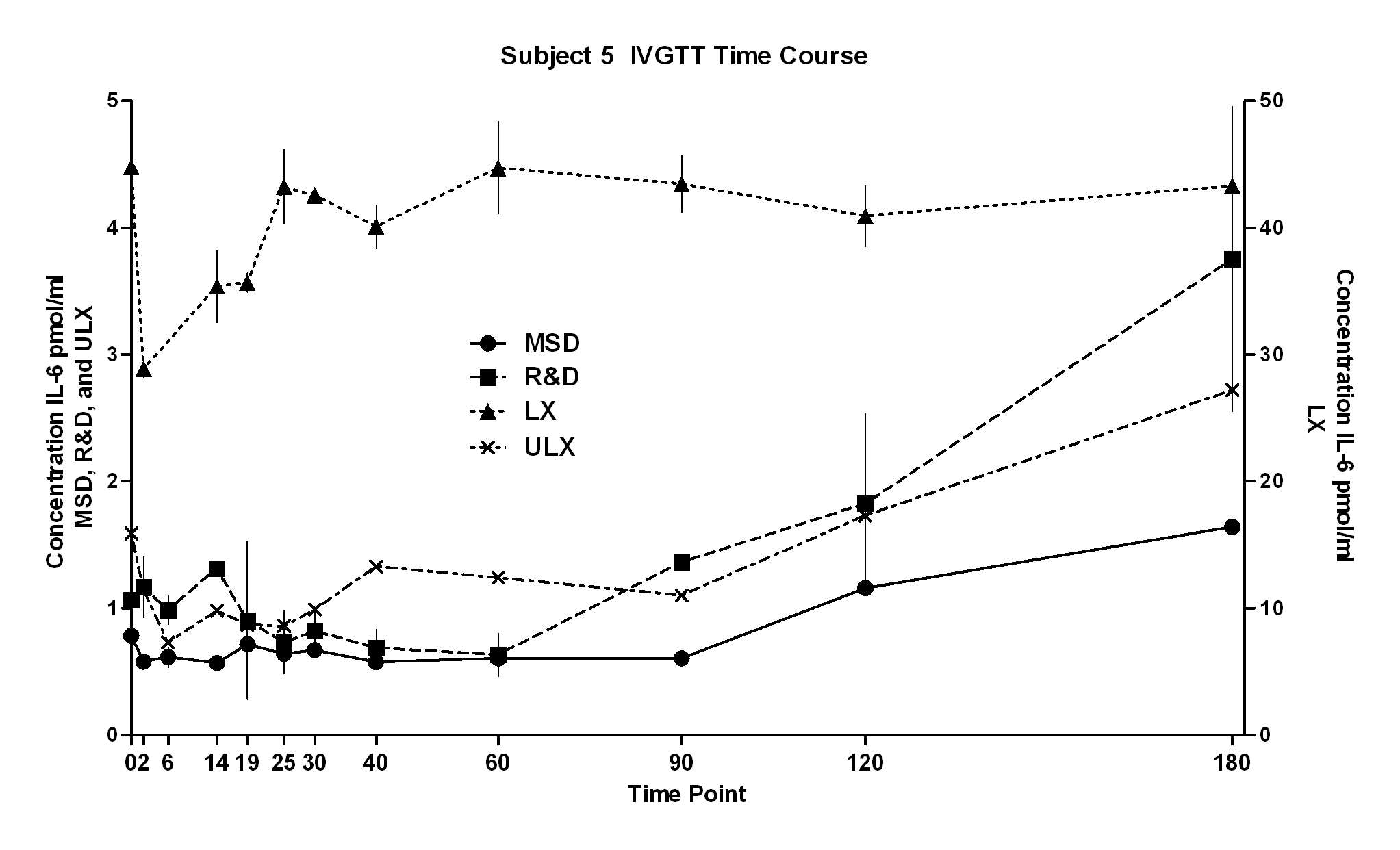

Supplement: Figure S5 — Plasma IL-6 during frequently sampled intravenous glucose tolerance test (IVGTT) in subject 5. Plasma concentrations of IL-6 were measured by MesoScale Discovery (• MSD), R&D High Sensitivity ELISA (▪ R&D), and Invitrogen Luminex (▴ LX) and Invitrogen Ultrasensitive Luminex (×ULX). Due to limited sample volumes, it was not possible to provide measurements for one time point (6 minutes) using LX. (TIF) [file pone.0030659.s005.tif]

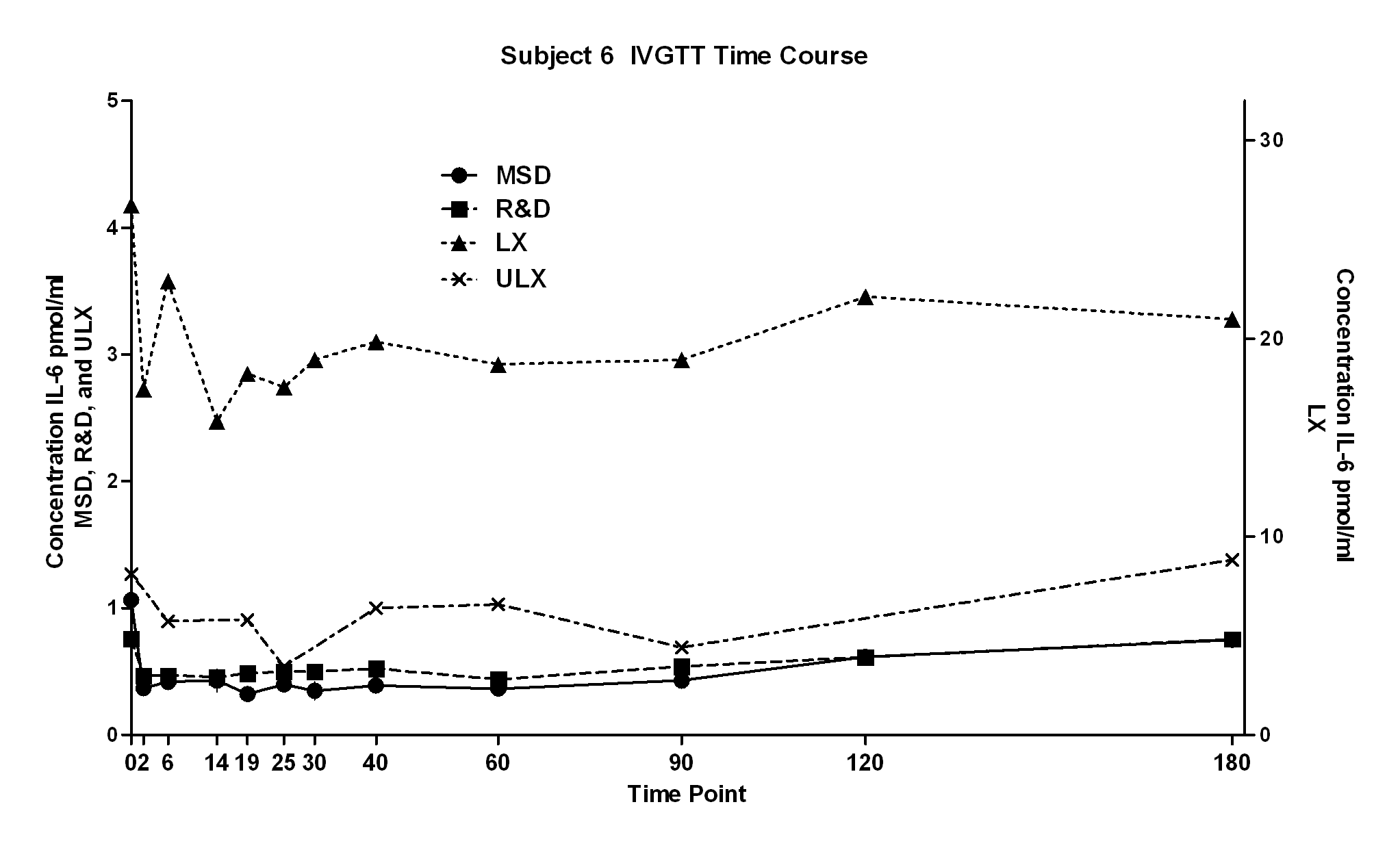

Supplement: Figure S6 — Plasma IL-6 during frequently sampled intravenous glucose tolerance test (IVGTT) in subject 6. Plasma concentrations of IL-6 were measured by MesoScale Discovery (• MSD), R&D High Sensitivity ELISA (▪ R&D), and Invitrogen Luminex (▴ LX) and Invitrogen Ultrasensitive Luminex (×ULX). Due to limited sample volumes, it was not possible to provide measurements for four time points (2, 14, 30, and 120 minutes) using ULX. (TIF) [file pone.0030659.s006.tif]

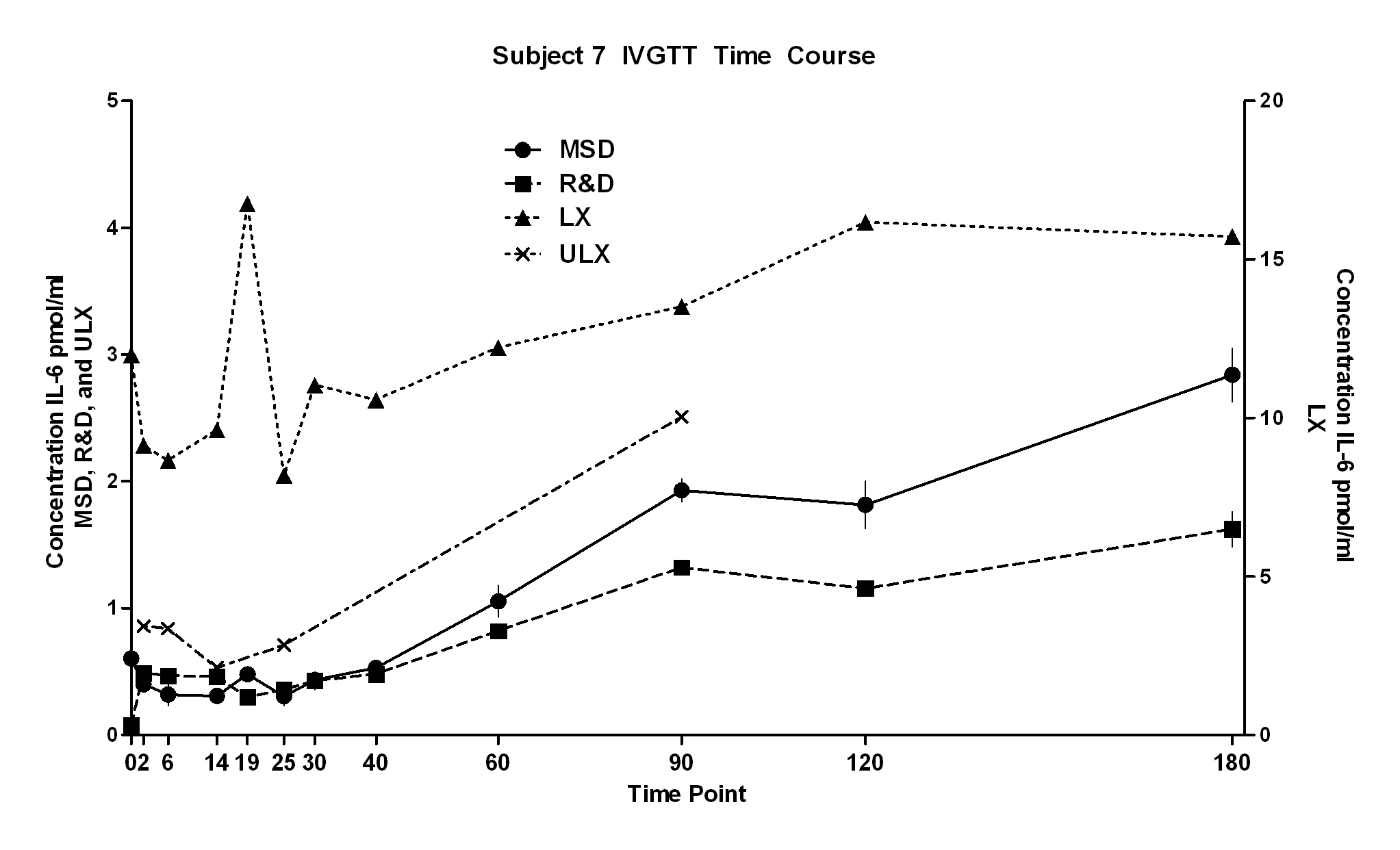

Supplement: Figure S7 — Plasma IL-6 during frequently sampled intravenous glucose tolerance test (IVGTT) in subject 7. Plasma concentrations of IL-6 were measured by MesoScale Discovery (• MSD), R&D High Sensitivity ELISA (▪ R&D), and Invitrogen Luminex (▴ LX) and Invitrogen Ultrasensitive Luminex (×ULX). Due to limited sample volumes, it was not possible to provide measurements for six time points (19, 30, 40, 60, 120, and 180 minutes) using ULX. (TIF) [file pone.0030659.s007.tif]

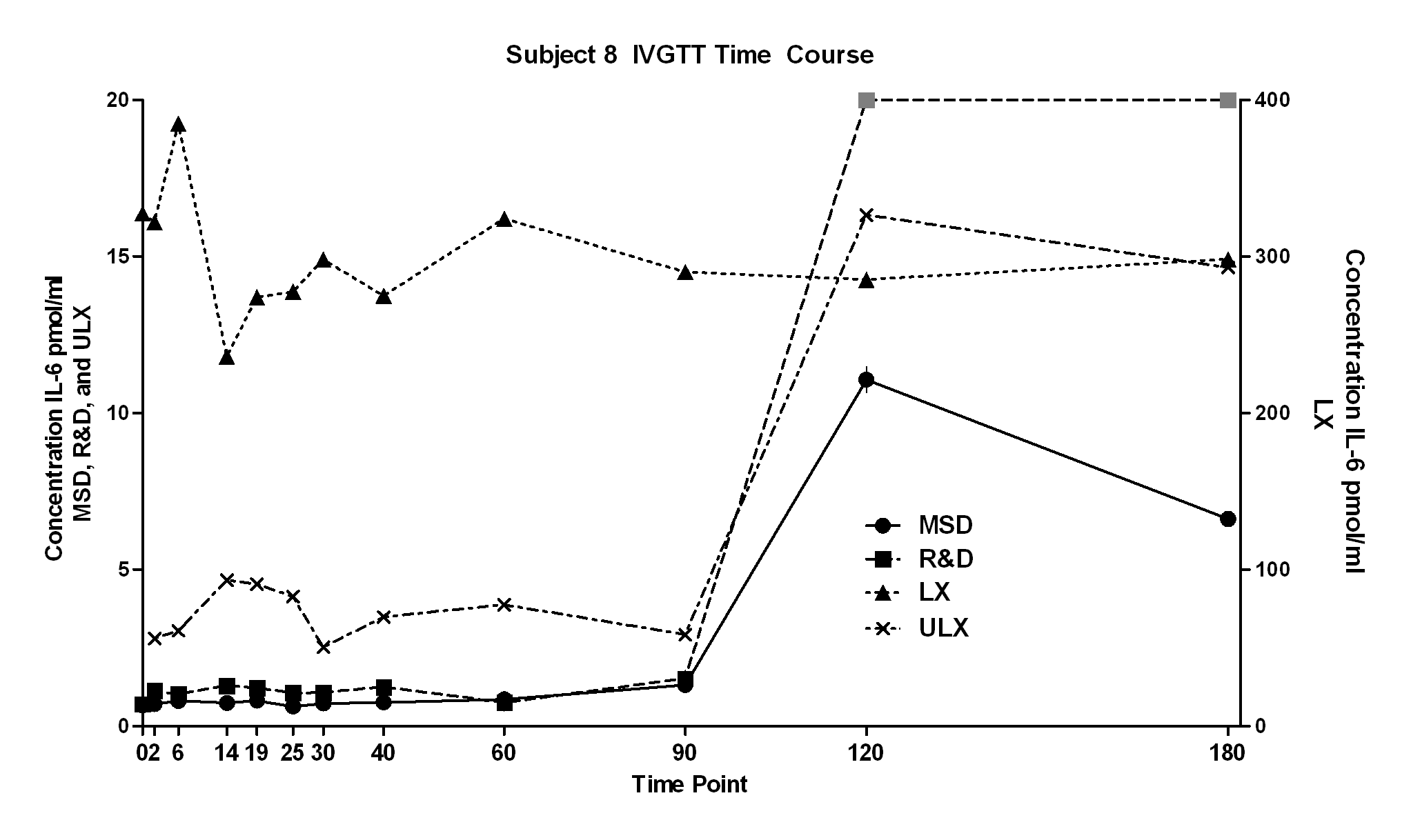

Supplement: Figure S8 — Plasma IL-6 during frequently sampled intravenous glucose tolerance test (IVGTT) in subject 8. Plasma concentrations of IL-6 were measured by MesoScale Discovery (• MSD), R&D High Sensitivity ELISA (▪ R&D), and Invitrogen Luminex (▴ LX) and Invitrogen Ultrasensitive Luminex (×ULX). Two samples returned IL-6 values above the range of detection (R&D) and were substituted with values twice the upper limit of quantification, as determined by the highest concentration of the standard curve and denoted by (<). (TIF) [file pone.0030659.s008.tif]

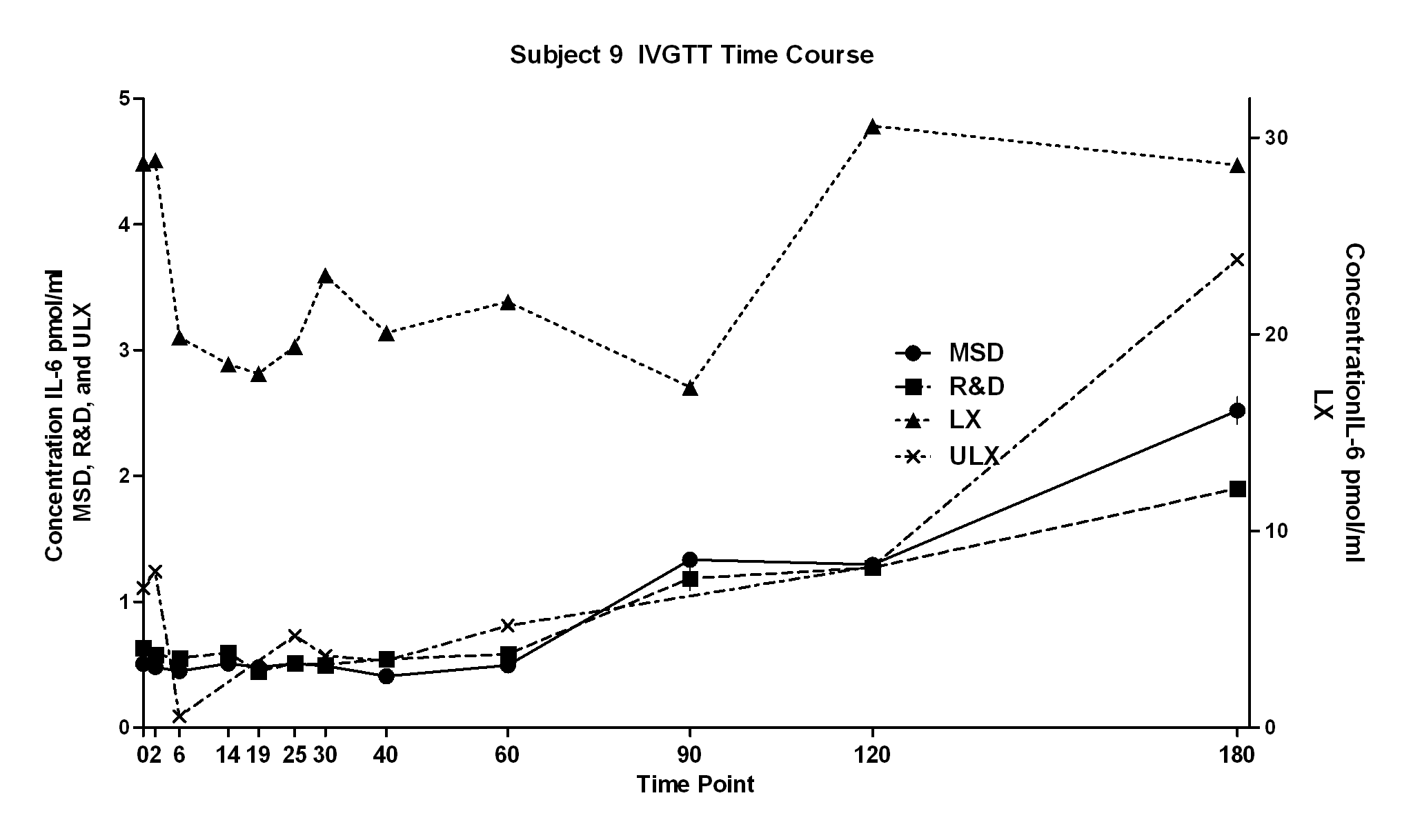

Supplement: Figure S9 — Plasma IL-6 during frequently sampled intravenous glucose tolerance test (IVGTT) in subject 9. Plasma concentrations of IL-6 were measured by MesoScale Discovery (• MSD), R&D High Sensitivity ELISA (▪ R&D), and Invitrogen Luminex (▴ LX) and Invitrogen Ultrasensitive Luminex (×ULX). Due to limited sample volumes, it was not possible to provide measurements for three time points (14, 19, and 90 minutes) using ULX. (TIF) [file pone.0030659.s009.tif]

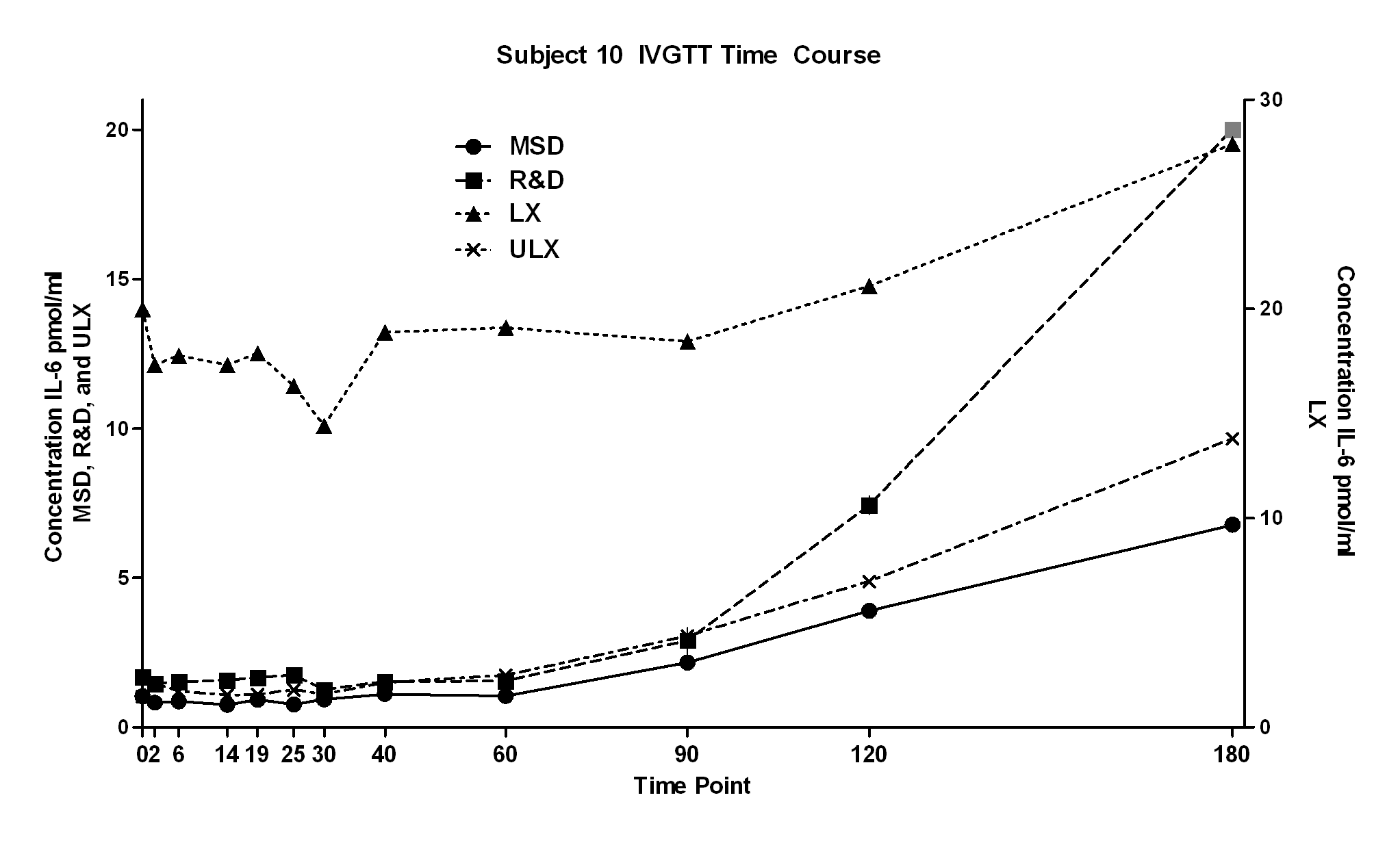

Supplement: Figure S10 — Plasma IL-6 during frequently sampled intravenous glucose tolerance test (IVGTT) in subject 10. Plasma concentrations of IL-6 were measured by MesoScale Discovery (• MSD), R&D High Sensitivity ELISA (▪ R&D), and Invitrogen Luminex (▴ LX) and Invitrogen Ultrasensitive Luminex (×ULX). One sample (180 minutes) returned an IL-6 value above the range of detection (R&D) and was substituted with a value twice the upper limit of quantification, as determined by the highest concentration of the standard curve, and denoted by (<). (TIF) [file pone.0030659.s010.tif]

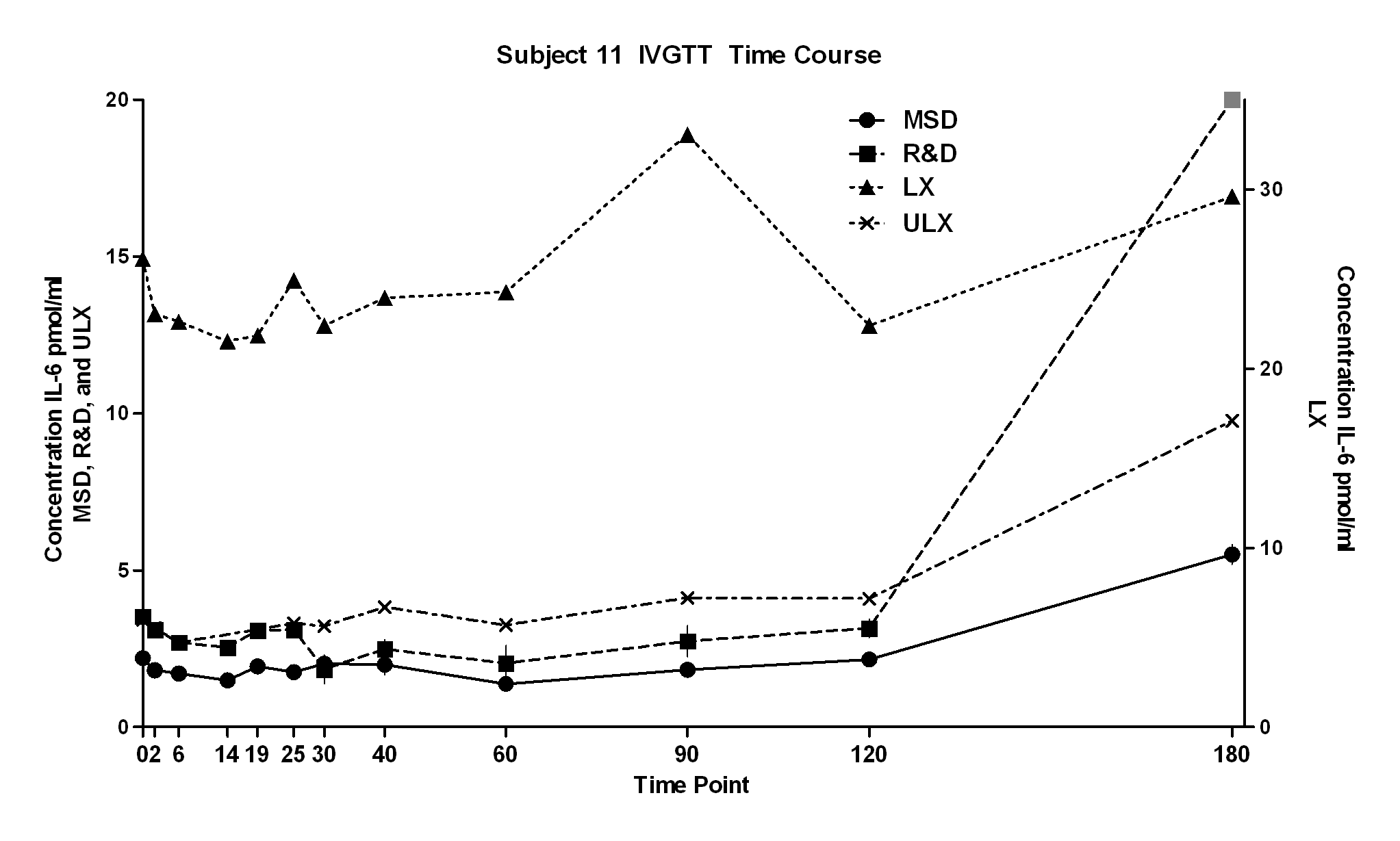

Supplement: Figure S11 — Plasma IL-6 during frequently sampled intravenous glucose tolerance test (IVGTT) in subject 11. Plasma concentrations of IL-6 were measured by MesoScale Discovery (• MSD), R&D High Sensitivity ELISA (▪ R&D), and Invitrogen Luminex (▴ LX) and Invitrogen Ultrasensitive Luminex (×ULX). One sample (180 minutes) returned an IL-6 value above the range of detection (R&D) and was substituted with a value twice the upper limit of quantification, as determined by the highest concentration of the standard curve, and denoted by (<). Due to limited sample volumes, it was not possible to provide measurements for one time point (14 minutes) using ULX. (TIF) [file pone.0030659.s011.tif]

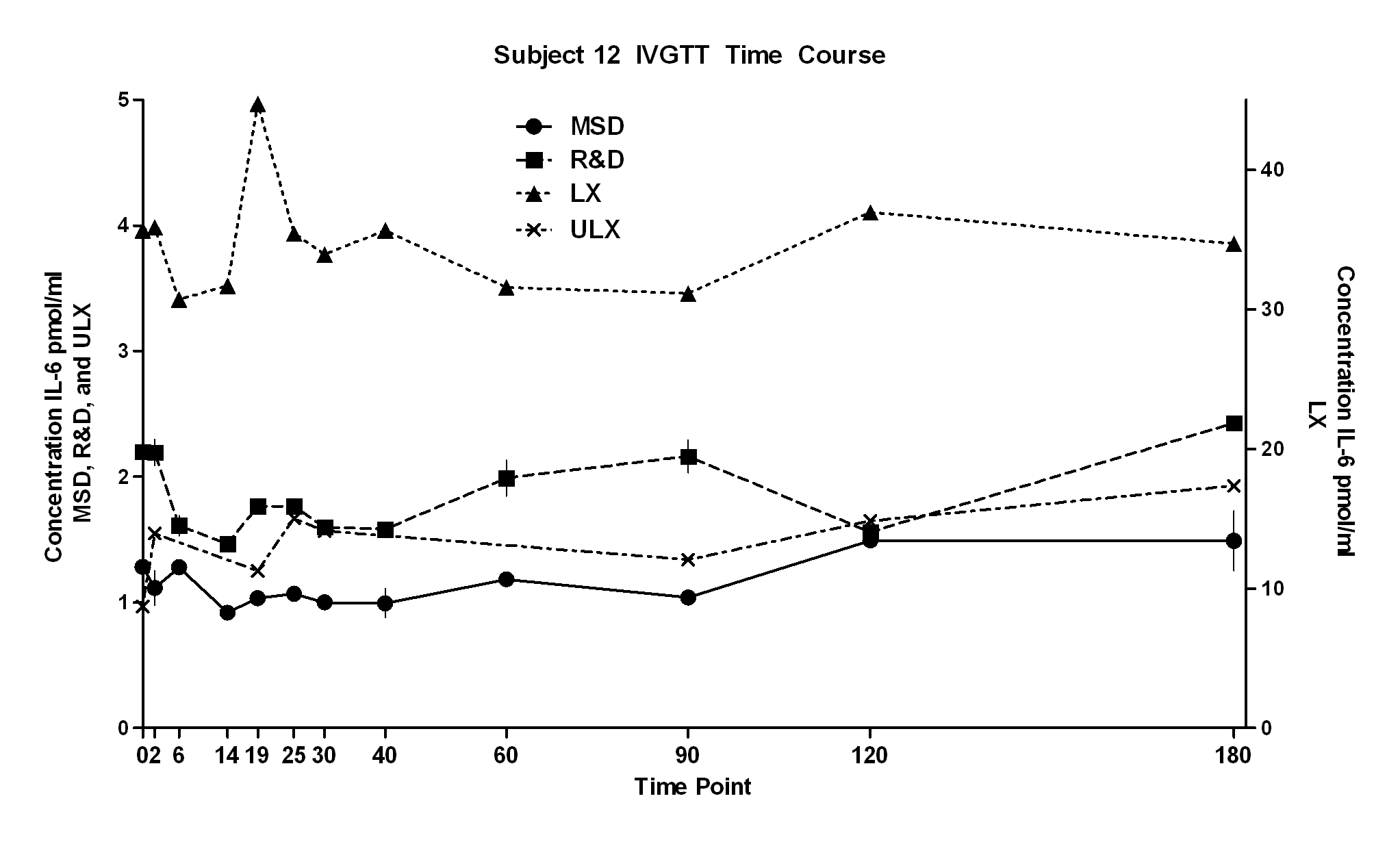

Supplement: Figure S12 — Plasma IL-6 during frequently sampled intravenous glucose tolerance test (IVGTT) in subject 12. Plasma concentrations of IL-6 were measured by MesoScale Discovery (• MSD), R&D High Sensitivity ELISA (▪ R&D), and Invitrogen Luminex (▴ LX) and Invitrogen Ultrasensitive Luminex (×ULX).Due to limited sample volumes, it was not possible to provide measurements for four time points (6, 14, 40, and 60 minutes) using ULX. (TIF) [file pone.0030659.s012.tif]

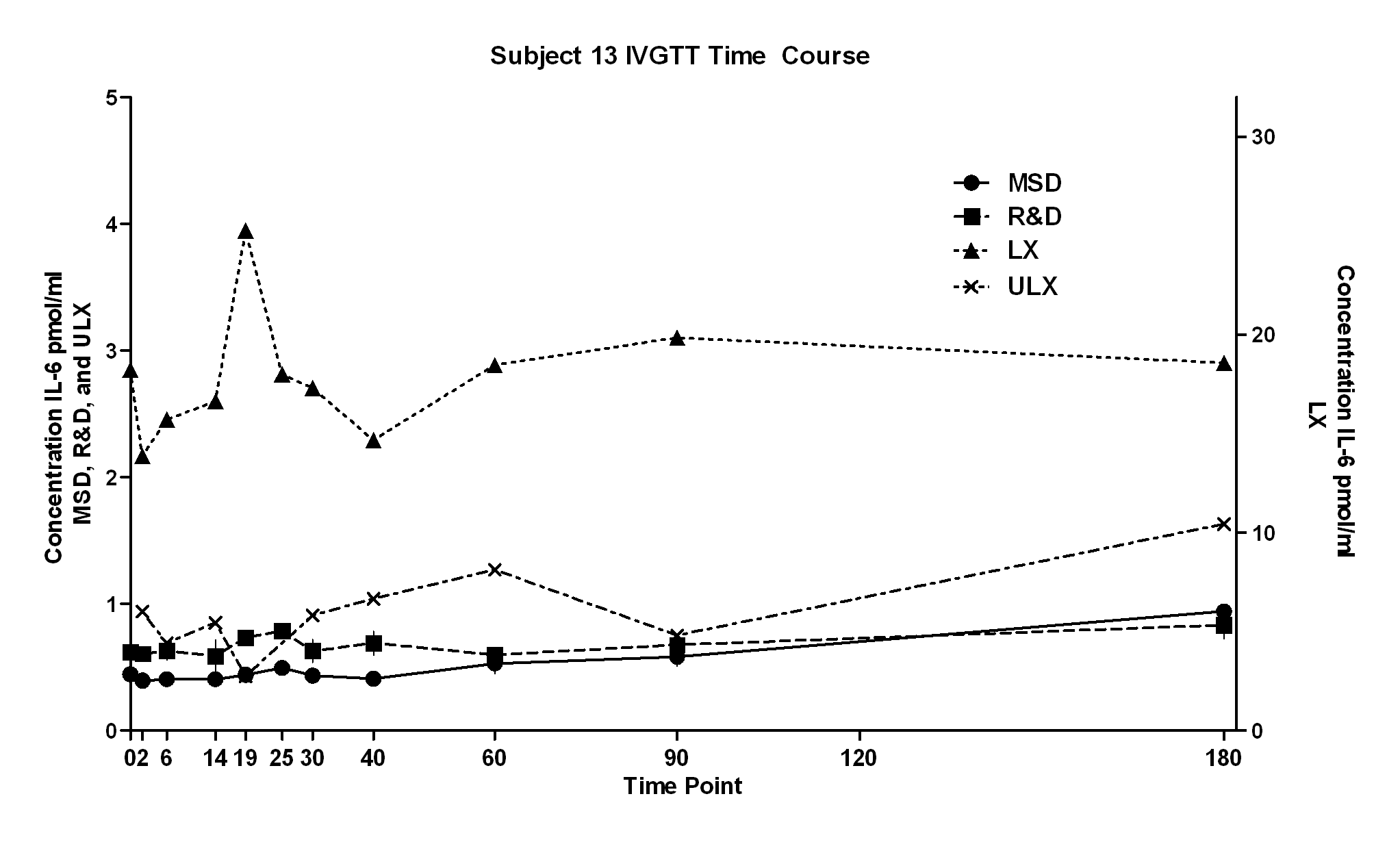

Supplement: Figure S13 — Plasma IL-6 during frequently sampled intravenous glucose tolerance test (IVGTT) in subject 13. Plasma concentrations of IL-6 were measured by MesoScale Discovery (• MSD), R&D High Sensitivity ELISA (▪ R&D), and Invitrogen Luminex (▴ LX) and Invitrogen Ultrasensitive Luminex (×ULX). No sample was available for measurement at one time point (120 minutes). (TIF) [file pone.0030659.s013.tif]

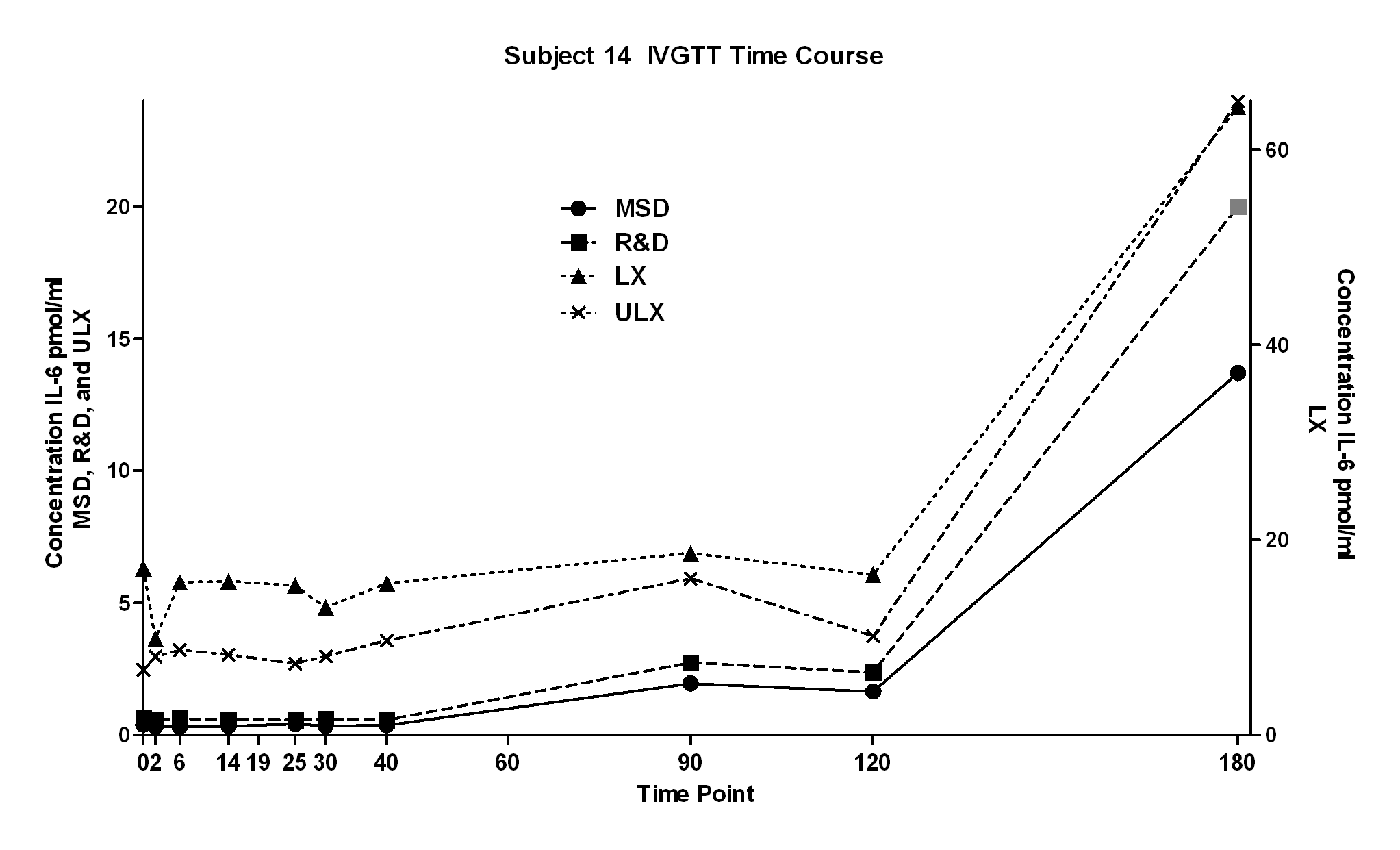

Supplement: Figure S14 — Plasma IL-6 during frequently sampled intravenous glucose tolerance test (IVGTT) in subject 14. Plasma concentrations of IL-6 were measured by MesoScale Discovery (• MSD), R&D High Sensitivity ELISA (▪ R&D), and Invitrogen Luminex (▴ LX) and Invitrogen Ultrasensitive Luminex (×ULX). One sample (180 minutes) returned an IL-6 value above the range of detection (R&D) and was substituted with a value twice the upper limit of quantification, as determined by the highest concentration of the standard curve, and denoted by (<). No sample was available for measurement at two time points (19 and 60 minutes). (TIF) [file pone.0030659.s014.tif]
